# Concurrent occurrence of acute bovine pulmonary edema and emphysema and endocardial fibroelastosis in cattle: A case history and literature review

**DOI:** 10.14202/vetworld.2018.971-976

**Published:** 2018-07-21

**Authors:** W. M. Hananeh, Z. B. Ismail

**Affiliations:** 1 Department of Pathology and Public Health, Faculty of Veterinary Medicine, Jordan University of Science and Technology, P. O. Box, 3030, Irbid 22110, Jordan; 2 Department of Clinical Veterinary Sciences, Faculty of Veterinary Medicine, Jordan University of Science and Technology, P. O. Box, 3030, Irbid 22110, Jordan

**Keywords:** bovine, cardiac anomaly, edema, emphysema, lungs

## Abstract

**Aim:**

The aim of this study was to describe the clinical and pathological findings of acute bovine pulmonary edema and emphysema (ABPEE) and left endocardial fibroelastosis in an adult dairy cow. In addition, a review of recent literature of these two conditions is provided.

**Materials and Methods:**

Necropsy and histopathological examination were performed using conventional techniques. A review of the literature was carried out using internet search engines such as PubMed and Google Scholar. Only published papers in scientific and refereed journals were reviewed.

**Results:**

Concurrent pathologies of ABPEE and left endocardial fibroelastosis were described in an adult Holstein Friesian cow. A review of recent literature concerning ABPEE and endocardial fibroelastosis revealed seven and two scientific reports of these conditions in cattle, respectively.

**Conclusion:**

Although rare, combined pathologies involving multiple organs such as the lungs and heart can be diagnosed in animals on careful clinical and histopathological examinations.

## Introduction

Acute bovine pulmonary edema and emphysema (ABPEE) is a well-recognized condition in cattle [1]. It occurs naturally throughout the year, primarily in late summer to fall after a sudden change in the diet or after exposure to certain toxins [1]. The condition is characterized by edema and emphysema of the pulmonary tissue leading to acute respiratory distress. It has been experimentally induced in cattle and goats [2,3]. The disease is considered non-contagious with the formation of pneumotoxic metabolites in the rumen as the main causative factor [2]. Other contributing factors may include nutritional additives, fungi, parasites, heat stress, viral or bacterial pneumonia, irritating gases, and hypersensitivity reactions [1-3]. The disease may occur in peracute, acute, or chronic forms and mostly affects animals over 2 years of age [1-3]. The morbidity and mortality rates can reach up to 50% and 10%, respectively [1-3]. The diagnosis is based on the history involving sudden dietary changes to a lush green pasture, the presence of acute respiratory distress signs, and lung lesions characteristic of severe edema and emphysema [1]. Treatment is usually not recommended, and the removal of cattle from the offending pasture may not prevent further cases from occurring [1].

Management of the disease is centered around prevention and control practices such as limiting access to offending pastures, diluting the effect of offending pasture by feeding hay before turn out, gradually increasing exposure to the pasture over time, using pastures before they become lush, and initially grazing pastures with less susceptible stock (cattle <15 months old or sheep) [1-3]. Feeding of monensin or lasalocid may help in preventing clinical cases by inhibiting the bacteria that convert l-tryptophan to 3-methylindole (3-MI). It is recommended to feed monensin for 1 day before introduction to offending pastures [1-3]. Lasalocid is recommended to feed for 6 days before introduction to offending pastures pretreatment period [1-4].

Endocardial fibroelastosis is a rare congenital cardiac anomaly that is characterized by dilation and expansion of the endocardium with fibrous and elastic tissue formation [5-10]. This may result in thickening of the endocardium that restricts ventricular filling and impairs valvar function. It has been reported mostly involving the left side of the heart, resulting in left-sided heart failure [5-11]. Endocardial fibroelastosis has been reported in different animal species including cats, dogs, horses, chickens, rabbits, and calves [4-11].

The diagnosis of a combined pathology of ABPEE and endocardial fibroelastosis in an adult dairy cow has not been reported before. In this study, concurrent pathologies of ABPEE and left endocardial fibroelastosis were described in an adult Holstein Friesian cow. In addition, a review of recent literature concerning ABPEE and endocardial fibroelastosis revealed only seven and two scientific reports of these two conditions in cattle, respectively.

## Materials and Methods

### Ethical approval

All procedures performed in this study were approved by the Jordan University of Science and Technology Animal Care and Use Committee.

### Gross and histopathological examination

Both the lungs and heart from a dead cow were presented for pathological evaluation to the Veterinary Health Center at Jordan University of Science and Technology. A board-certified pathologist carried out the gross examination. Representative tissue samples from the heart and lungs were fixed in 10% buffered formalin for 24 h. Then, the tissues were processed and embedded in paraffin wax using the routine procedure. 4-5 μm thick sections of the heart and lungs were made and stained by hematoxylin and eosin (H and E) stain. The stained slides were examined by light microscope.

### Review of recent literature

A review of the literature was carried out using internet search engines such as PubMed and Google Scholar. All published papers related to ABPEE and endocardial fibroelastosis in cattle between the years 2000 to 2018 were extracted and reviewed. Only published papers in scientific and refereed journals were considered.

## Results

### History and gross pathology findings

The cow was an adult Holstein Friesian dairy cow. According to the owner, the cow was found dead without showing any abnormal signs. The owner did not report any changes in diet or feeding management in the few days before the death of this cow. The cow was housed along with 19 others in a closed system. None of the cows showed any abnormal clinical signs.

**Figure-1:**
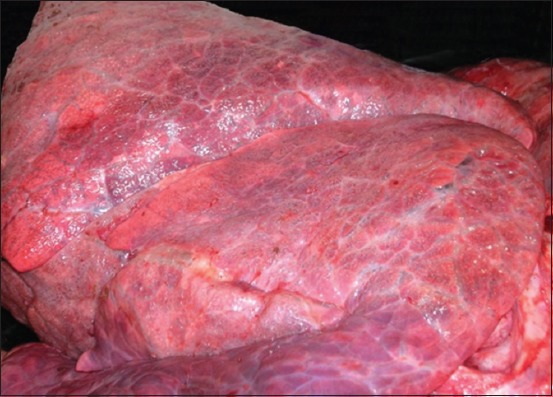
Acute bovine pulmonary edema and emphysema, lung, bovine. Lungs were heavy and failed to collapse with variably sized gas bubbles throughout subpleura and interlobular fascia.

**Figure-2:**
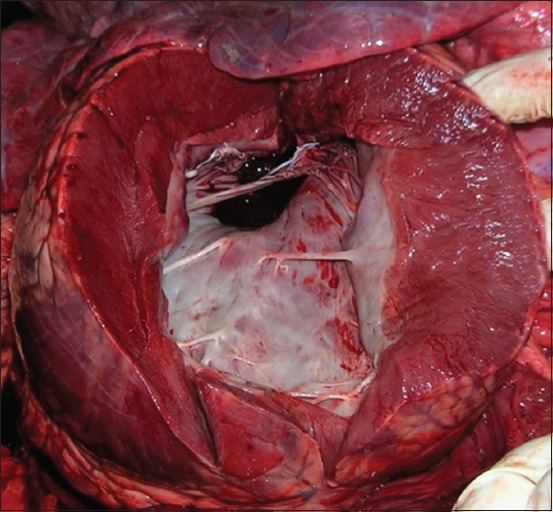
Left endocardial fibroelastosis, heart, bovine. The endocardium of left ventricle, as well as the chordae tendineae, was diffusely and uniformly whitish and opaque with a smooth endocardial surface.

Necropsy examination was performed by a field veterinarian. The veterinarian reported that, on necropsy examination, postmortem autolysis was mild. Both the lungs were heavy and failed to collapse with variably sized gas bubbles throughout subpleura and interlobular fascia that extended to the mediastinum (Figure-1). Diffusely, the lungs were dark and congested with severe pulmonary edema that extensively widened the interlobular fascia. Mediastinal lymph nodes were edematous. Trachea and bronchi were filled with a large amount of white frothy edema fluid. The pericardium was previously cut. The endocardium of the left ventricle as well as the chordae tendineae was diffusely and uniformly whitish and opaque with a smooth endocardial surface (Figure-2). No other abnormalities were seen elsewhere.

### Histopathological findings

**Figure-3:**
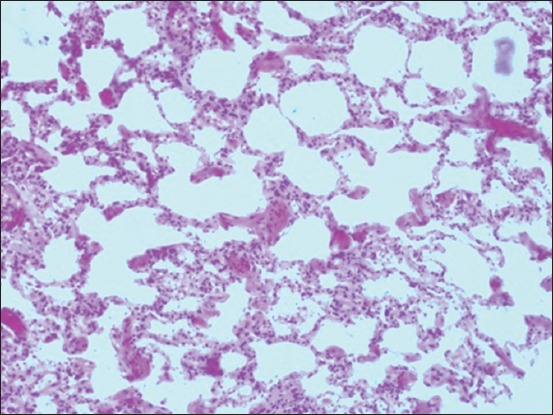
Acute bovine pulmonary edema and emphysema, lung, bovine. The alveolar walls were moderately thickened by prominent fibromusculature, edema, and a few inflammatory cells. In multiple areas, the alveolar wall was ruptured where multiple alveolar spaces were communicated with each other (H and E stain).

**Figure-4:**
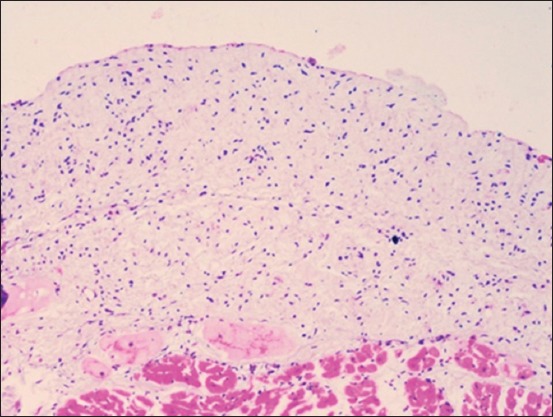
Left endocardial fibroelastosis, heart, bovine. The left endocardium was markedly and uniformly thickened up to 10× normal thickness caused by abundant connective tissues primarily, elastic fibers (H and E stain).

Histopathologically, the pulmonary parenchyma was diffusely disrupted by abundant edema and numerous variably sized gas bubbles. They were filling more than 90% of the alveolar spaces, interlobular septa, and subpleura. The alveolar walls were moderately thickened by prominent fibromusculature, serofibrinous edema, and a few inflammatory cells primarily neutrophils (Figure-3). Mild fibrinous material and increased alveolar macrophages were seen in multiple alveolar spaces. In multiple areas, the alveolar wall was ruptured and alveolar spaces were communicated with each other. The left endocardium was markedly and uniformly thickened up to 10× normal thickness caused by abundant connective tissues primarily, elastic fibers (Figure-4). These fibers were loosely arranged in multiple layers with moderate edema that separated the fibers. In multiple areas, the fibers breached the endocardium into the underlying myocardium causing widening the edematous interstitium. The lymphatic vessels were dilated.

### Review of recent literature

A review of recent literature concerning ABPEE and endocardial fibroelastosis revealed six and two scientific reports of these two conditions in cattle, respectively (Tables-1 and 2).

**Table-1:** Review of the history, etiology, clinical signs, gross, and histopathological data of ABPEE in cattle.

History/etiology/clinical signs	Gross and histopathology	References
Feedlot cattle	Interstitial pneumonia	[12]
Acute respiratory distress		
High plasma 3-methylindole		
No bovine respiratory syncytial virus antigen was detected in affected lungs		
Feedlot calves	Interstitial pneumonia with multifocal to coalescing distribution	[10]
Acute respiratory distress		
	Suppurative bronchopneumonia	
Non-significant contribution of bovine respiratory syncytial virus infection	Bronchiolitis fibrosa obliterans	
Dairy buffalo (*Bubalus bubalis*)	No gross and histopathological lesions reported	[13]
Acute respiratory distress		
Sudden shift from Berseem (*Trifolium alexandrinum*) to *Brassica juncea* fodder		
Subcutaneous emphysema		
Normal rectal temperature		
Adult dairy cows of Holstein and Brown Swiss breed	Dark red not collapsed a lung	[14]
	Marked interlobular emphysema	
Acute respiratory distress		
	Alveolar and interlobular emphysema	
Sudden transfer from mature and dry pasture to another luxurious sprouting one	Congestion and edema	
	Hyaline degeneration of the alveolus wall	
Subcutaneous emphysema		
	Moderate diffuse infiltration of macrophages and eosinophils	
Case-control study	Bronchiolitis	[15]
Feedyard cattle	Bronchopneumonia	
Acute respiratory distress		
Rectal temperature was greater in ABPEE		

ABPEE=Acute bovine pulmonary edema and emphysema

**Table-2:** Review of the history, etiology, clinical signs, gross, and histopathological data of endocardial fibroelastosis in cattle.

History/etiology/clinical signs	Gross and histopathology	References
A 14-year study of calves with cardiac defects 3 calves	Endocardial fibroelastosis with calcification	[16]
5-month-old Holstein Heifer	Multifocal areas of thickening and opacification of the endocardium of the left and right ventricles	[17]
Weight loss, abdominal distention, dyspnea, and decreased appetite		
Systolic murmur	Excessive elastic fibers formation in the endocardium consistent	
Tricuspid valve insufficiency		
Right-sided heart failure		

### ABPEE

In one study involving five large feedlots in West Central Saskatchewan, the nature and distribution of histopathological lesions in the lungs of feedlot cattle with interstitial pneumonia were described previously [12]. The association of bovine respiratory syncytial virus (BRSV) was also established in one study in feedlots [12]. The lesions were characterized by interstitial pneumonia of the dorsal portions of caudal lung lobes with multifocal to coalescing distribution in most of the cases [12]. Hyaline membrane formation and type II alveolar epithelial cell hyperplasia were present in acute and subacute cases in addition to concurrent bronchopneumonia [12]. Chronic cases were characterized by bronchitis, bronchiolitis, and bronchiolitis fibrosa obliterans [12]. The BRSV antigen was detected in only 2 of 28 cases using immunohistochemistry [12]. BRSV was found associated with severe bronchiolar epithelial necrosis in these cattle [12].

In another field study involving 38 cattle that were slaughtered because of acute interstitial pneumonia (AIP), 3MI plasma concentrations were significantly elevated in cattle affected with AIP compared to normal pen-mates [13]. However, 3MI concentrations in lung tissue and microsomal protein did not differ significantly between the two groups. Furthermore, 3MI was not detected in rumen fluid obtained from either group [13]. There was also a non-significant difference in the type and number of bacterial and protozoal populations in the rumen in the two groups, although fewer numbers of cellulolytic bacteria were detected in AIP-acted cattle [13]. In this group of AIP cattle, BRSV antigen was not detected in any of the lung tissues [13].

In dairy buffalo, the occurrence of acute pulmonary emphysema and edema was described after a sudden change of the diet from Berseem (*Trifolium alexandrinum*) to *Brassica juncea* fodder [14]. Clinical signs were characterized by tachypnea, dyspnea, expiratory grunt, crackles, and subcutaneous emphysema [14]. The rectal temperature was normal in all affected cattle that characterized the condition clinically [14]. The authors reported remarkable improvement after treatment using prednisolone, dexamethasone, diclofenac sodium, and furosemide [14]. In addition, chlortetracycline was administered orally to affected cattle [14]. No deaths were reported in this group of cattle, and all affected animals were recovered completely within 10 days [14].

The epidemiological, clinical, gross, and histological lesions associated with acute pulmonary edema and emphysema in cattle were reported in four outbreaks in the states of Parana and Santa Catarina of Brazil [15]. The disease occurred spontaneously following the introduction of cattle to luxurious sprouting pasture [15]. All animals were adult dairy cows of Holstein and Brown Swiss breed. Clinical signs were characterized by dyspnea, extended head and neck, open mouth breathing, and subcutaneous emphysema [15]. The lungs of dead animals failed to collapse and were dark red with marked interlobular emphysema [15]. On histology, the lesions were characterized by alveolar and interlobular emphysema, congestion and edema, hyaline degeneration and diffuse infiltration of macrophages, and eosinophils [15]. The disease could be reproduced experimentally in one cow by oral administration of L-tryptophan in a single dose [15]. Similar clinical signs, gross, and histopathological lesions to those reported in naturally occurring disease were detected in this animal [15]. It is worth mentioning that hyaline membrane formation is a characteristic lesion in ABPEE; however, in this case, it was not seen in the examined lung sections. Hyaline membrane formation within alveoli is seen in animals that survive for several days. In this case, the animal died suddenly.

In a case-control study, different management and physiological factors that may contribute to AIP in feedlot cattle were studied [18]. Affected animals and normal pen-mates were subjected to antemortem and postmortem examinations [18]. In this study, the rectal temperature and rumen pH values in AIP cattle were higher than normal pen-mates. Bronchiolitis and bronchopneumonia were evident in most of the affected animals. The authors reported no association between AIP and feed intake patterns, serum amylase, or lipase levels [18].

An outbreak of AIP in Angus cattle was recently described [16]. The duration of the disease was approximately 3 days. The outbreak occurred in cattle after feeding on wafers by-products from a nearby food factory [16]. The clinical signs were dyspnea and abdominal breathing. In this study, grossly the lungs were dark red, firmer than normal, and failed to collapse with significant interlobular edema [16]. On histopathology, there was thickening of interlobular pulmonary septa, interstitial edema and emphysema, inflammatory cell infiltration, type II pneumocyte proliferation, and thickening of the alveolar walls [16].

In a case-control study, different management and physiological factors that may contribute to AIP in feedlot cattle were studied [17]. Affected animals and normal pen-mates were subjected to antemortem and postmortem examinations [17]. In this study, the rectal temperature and rumen pH values in AIP cattle were higher than normal pen-mates. Bronchiolitis and bronchopneumonia were evident in most of the affected animals. The authors reported no association between AIP and feed intake patterns, serum amylase, or lipase levels [17].

### Endocardial fibroelastosis

Endocardial fibroelastosis with calcification was reported in three calves as a part of a long (14 years) study of calves with different cardiac anomalies [18]. In another study, endocardial fibroelastosis was diagnosed in a 5-month-old Holstein Heifer that had clinical signs of tricuspid valve insufficiency [18]. Endocardial fibroelastosis in this calf was diagnosed based on histopathological evidence of excessive elastic fibers consistent with fibroelastosis of the endocardium observed after applying special stains [18]. Clinically, the calf suffered from weight loss, abdominal distention, dyspnea, and decreased appetite for 3 weeks before euthanasia [18]. Right-sided heart failure and a systolic murmur were also evident on physical examination. Cardiac catheterization and echocardiography confirmed the tricuspid valve insufficiency and right-sided heart failure. Grossly, examination of the heart revealed areas of multifocal thickening and opacification in the endocardium of the left and right ventricles [18].

## Discussion

Based on the clinical history, gross and microscopic findings of diffuse pulmonary congestion, edema, and emphysema, a diagnosis of ABPEE was determined. Peracute enzootic pneumonia was considered one of the differential diagnoses in this case [1]. However, enzootic pneumonia occurs primarily in young beef cattle and housed dairy calves and was excluded. Another differential that was also considered is BRSV which can produce synergistic effects with 3MI when both are present in an animal [1]. BRSV could have been ruled out by virus isolation in this case. Parasitic lung infestation with a large number of *Dictiocaulus* sp. larvae in susceptible animals may also produce a reaction that causes acute pulmonary emphysema similar to the one seen here in this case [1]. Parasitic pneumonia, however, is not known to occur in the geographic area where this cow comes from and therefore was excluded.

While ABPEE is considered common in cattle, endocardial fibroelastosis is a rare anomaly. Further, the combination of these two conditions is extremely unusual, yet, in this study, a combined pathology is described in an adult dairy cow for the first time. The gross and histopathological findings from both the lungs and heart were indicative of concurrent ABPEE and endocardial fibroelastosis. This case was considered unique because of several issues. This was the first case that experienced the two conditions together. Second, it was the only cow that was affected among 20 animals in the herd. Third, the cow experienced a very rapid clinical course. Finally, this cow was household. In one report, ABPEE was reported previously in a yearling bullock that was household [19]. The bullock was reportedly switched from a pasture of grass and barley to another one with lush silage. In our case reported here, the owner stated that the cow had died suddenly; it was believed that the animal died because of hypoxia that was occurred rapidly. This was supported by the presence of severe lesions in the lungs and heart. The severity of edema and numerous gas bubbles alone would be enough to create severe hypoxia and rapid death of the animal. It was reported that respiratory signs of ABPEE occur suddenly and death could occur within 48 h of the initial clinical signs [20]. In this case, the death occurred within 24 h as the owner claimed.

The sudden death of two cases of thoroughbred horses was reported the following exercise [6]. Equine endocardial fibroelastosis was diagnosed in these two animals [6]. No other gross or histopathological changes were found within the affected equine hearts other than the fibroelastosis. In this case, sudden death might be due to severe respiratory compromise exacerbated by the endocardial lesions of the left ventricle. In one outbreak of atypical interstitial pneumonia affecting 15 Angus cattle aged 7–30 months in South Rio Grande do Sul, 9 animals were dead within 24 h [16]. A fatal case of ABPEE in a 5-year-old Bazadaise cross was reported after being moved to lush pasture [21].

Sudden death due to endocardial fibroelastosis was also reported in cats [22]. There are no known predisposing factors to this condition other than it is a rare congenital condition. It was reported that respiratory infections in cats hasten the onset of endocardial fibroelastosis [22]. In this case, a similar scenario could be applied. Furthermore, in dogs, chronic experimental impairment of cardiac drainage resulted in the increased amount of fibrous and elastic connective tissue within the endocardium [23]. Endocardial fibroelastosis was also reported following subendocardial edema due to the obstruction of cardiac lymphatics [22]. For better understanding, the mechanism of endocardial fibroelastosis in human, different animal models were developed [24,25]. It was reported two key regulators of endocardial fibroelastosis development [24]. Those factors were immaturity and lack of intracavitary blood flow.

## Conclusion

In this study, a review of scientific literature concerning ABPEE and endocardial fibroelastosis was performed. Although ABPEE is commonly diagnosed in both housed and pastured cattle, endocardial elastosis is rarely diagnosed in clinical practice. Uniquely, in this review, we described the gross and histopathological findings in a case where these two conditions occurred concurrently.

## Authors’ Contributions

WMH diagnosed the case and wrote the pathology part of the manuscript. ZBI completed the rest of the manuscript. Both authors read, finalized, and approved the manuscript.
